# Effectiveness of Body Remodeling and Cellulite Appearance Improvement Treatments in the Thighs Using Symmed Radiofrequency Device

**DOI:** 10.1111/jocd.16796

**Published:** 2025-01-15

**Authors:** Alejandra Fernández Santos, Alejandra Iglesias Fernández, Lorena Suárez Fernández, Leticia Huergo Zapico, Susana Valero Freitag

**Affiliations:** ^1^ Termosalud SL Gijón Spain

**Keywords:** cellulite, collagen, radiofrequency, skin structure, subcutaneous fat

## Abstract

**Background:**

Aging associated with the passage of time causes alterations in subcutaneous tissues and the overall appearance of the skin that generate aesthetic unconformities in people. Within the wide range of available techniques on the market, radiofrequency (RF) diathermy has emerged as one of the most used non‐invasive methods to combat them.

**Objective:**

This clinical trial aims to determine and quantify the effectiveness and safety of the high‐frequency RF‐based device Symmed (Termosalud Inc., Gijón, Spain) in cellulite appearance improvement and thigh remodeling treatments.

**Material and Methods:**

Eight sessions of Symmed were performed every 72/96 h in eight subjects' thighs. Safety and effectiveness of the device were evaluated before and after the treatments by digital photographs, body contours measurements, and ultrasound images of skin echogenicity and fat tissue thickness. Information regarding the satisfaction level of the participants was obtained throughout a customized questionnaire.

**Results:**

After the treatments, significant reductions of 3% in thighs circumferences (*p* < 0.05) and 20% in subcutaneous fat layer thickness (*p* < 0.01) were detected. Dermal echogenicity, used as an indicator of skin organization and collagen content, showed a significant 6% increase (*p* < 0.05). Before and after photographs revealed a general improvement in skin appearance. The results were complemented by a high satisfaction level (100%) and the absence of adverse events.

**Conclusion:**

Symmed RF therapy is a non‐invasive, safe, effective, and well‐tolerated procedure for the treatment of thighs cellulite appearance and body remodeling.

## 1 Introduction and Objective

Cellulite appearance, corporal fat accumulation, and other skin alterations (wrinkles, sagging, laxity, …) are common aesthetic concerns associated to the passage of time [1, 2]. Their occurrence can significantly impact an individual self‐perception and well‐being, resulting in people constantly seeking aesthetic treatments options [2]. According to scientific literature, the occurrence and the severity of these disconformities is directly linked to the interaction of internal (genetics, hormones, and metabolism) and external factors (UV exposure, physical activity, lifestyle, …) [1, 3, 4]. However, the greater influence of some of these factors causes the alterations not to appear with the same frequency in all body areas [3]. For example, among others, hormonal factors are related to the progressive redistribution of adipose tissue and the worsening of cellulite appearance in the lower limbs—buttocks and thighs‐ of women and the abdominal region of men [4]. Similarly, areas commonly affected by solar radiation, such as face, neck, forearms, or back of the hands exhibit visible signs of skin degeneration faster than other sites [1].

The exposure to the previous intrinsic and extrinsic factors generates multilayer internal alterations that are responsible for the aged appearance of our bodies [1, 5]. Generally, in the superficial layers of the skin, a damage in structural components and a decline of its biomechanical properties occur (loss of strength, elasticity, and hydration) [1, 5]. Between other processes, there is an alteration of the dermal extracellular matrix (ECM) elastin content [5], and a reduction, degradation, and disorganization of collagen bundles [1, 5]. In the subcutaneous fat tissue, aging induces different changes. Together with fat cells hypertrophy (increasing cell size) [6] and the fibrogenesis of hypodermal matrix [7], a redistribution of subcutaneous tissue is manifested [4].

Traditionally, invasive methodologies have been chosen to manage age‐associated changes, such as cellulite appearance or localized fat accumulation [8]. However, their high surgical risks and long recovery time have promoted the development of numerous non‐invasive techniques [8, 9]. Here, highlights the role of RF, the first choice option [9]. Non‐invasive RF treatments rely on the use of an electromagnetic current to induce a homogenous volumetric tissue heating [8]. In the tissues, high temperatures promote biological changes in skin and subcutaneous components to the synthesis and reorganization of dermal collagen matrix [2], as well as the mobilization of hypodermic fatty deposits [3]. In turn, thermal stimulation also enhances blood circulation, contributing to tissues oxygenation, movement of removed fat, and drainage of accumulated fluids [3, 10].

Previous studies have evaluated the effectiveness of RF systems as a tool to cellulite appearance reduction and body remodeling treatments [2, 9, 11]. Nevertheless, the devices used in these trials do not always stimulates all tissue layers [12]. Symmed (Termosalud Inc., Gijón, Spain) is a non‐invasive monopolar RF system developed to stimulate both the skin and underlaying tissues. This study was conducted to evaluate the effectiveness and safety of the Symmed RF device to non‐invasively treat skin problems and body fat accumulation as an alternative to traditional methods.

## 2 Materials and Methods

### 2.1 RF Device

Symmed is a monopolar and non‐invasive RF diathermy device. The system distributes a high‐frequency, low‐impedance alternating current through a circuit composed by the handpiece, the target tissues and a neutral plate. The device incorporates the alternative use of resistive and capacitive energy delivery modes to perform the treatments. In turn, it allows to adjust the energy output (up to 200 W) and modify procedures duration. Through a temperature sensor and a thermal optimization system the device ensures treatment safety and the enhancement of results.

### 2.2 Participants

The trial was approved by the Research Ethics Committee of the Principality of Asturias. The study considered eight healthy women from 35 to 57 years old and Fitzpatrick skin types II to III. Candidates were evaluated before starting the trial according to the study inclusion/exclusion criteria. Volunteers under the age of 18, over the age of 80, and manifesting any of the RF contraindications (S1) were excluded. Subjects between 18 and 80 years old exhibiting localized excess of adipose tissue and/or cellulite imperfections in the thighs were included. Written informed consent and photographic authorization were obtained from all the participants following a complete explanation of the study protocol. Participants were also asked to continue with their usual lifestyle. The flow of the participants throughout the trial was included in S2.

### 2.3 Treatment Design

The treatment was delivered in eight sessions of 30 min every 72/96 h. In each session, a high frequency RF current was applied to progressively increase thighs skin temperature until hyperthermia (41°C) was reached. This state was maintained for 10 min. Conductive cream was applied on the skin to facilitate the massage of the treated area with the electrode.

The evaluation of the device effectiveness and safety was performed by taking measurements (S3) at baseline (before the treatment) and 1 week after the last treatment session (follow‐up visit).

### 2.4 Body Circumference Evaluation

Circumference measurements were taken with a standardized measuring tape at 2 different heights of the left thigh. Subjects remained in a standardized standing position throughout the evaluation and the measure tape was kept constantly in contact with the subjects' skin. Upper contour (H1) was taken under the gluteus and lower contour (H2) was measured 5 cm above the popliteal fossa. To standardize the evaluation, coordinates of the measuring points were documented (S4).

### 2.5 Ultrasound Measurements

Ultrasound analysis of dermal echogenicity (DE) and subcutaneous adipose tissue thickness were made using the DermaLab Combo Cortex device (Cortex Technology, Denmark) at baseline and at the follow‐up session. Subcutaneous fat layer thickness was recorded using a 10 MHz ultrasound probe as the distance between the deep dermis and the deep fascia membrane. DE was evaluated with a 20 MHz focused ultrasound probe as an indirect indicator of dermal collagen content. Collagen is an abundant and a highly eco‐rich protein of the ECM that reflects ultrasound waves to a greater extent compared to surrounding structures. Therefore, by quantifying the echogenic changes (brightly areas), dermal collagen content increase can be indirectly assessed [11]. Ultrasound scans and the associated quantitative data were obtained at three measuring points of the treated region (S4).

### 2.6 Photographic Evaluation

Before and after treatment photographs were taken with a digital camera (CANON EOS 60D) in standardized position and lighting conditions. Subjects were positioned on a fixed floor template in front of a graduated panel with their arms flexed. Photographs from the front, right side, left side and back were taken. Before and after photographs were combined using Photoshop software (24.1.1 version). To visually evaluate the improvement, both photographs were identically transformed with the Photoshop software into inverted black and white images and adjusted in terms of illumination and contrast.

### 2.7 Safety and Satisfaction Assessment

Satisfaction evaluation was carried out 1 month after the final measurement session according to a questionnaire developed by Termosalud SL (S5). Questions regarding treatment procedure, compliance level and adverse effects occurrence were registered by selecting one pre‐established answer or according to a 0–10 numerical scale.

### 2.8 Statistical Analysis

The data of the study was analyzed using the R software (4.3.0 version) and the Rcmdr package (2.8‐0 version) for Windows. Descriptive analysis (means, medians, ranges and standard deviations) was performed to express the improvement in DE, adipose tissue thickness and body contours. Non‐normality was assumed for all the tests due to sample sizes smaller than 20 individuals. Comparisons before and after treatment were performed using the Wilcoxon's signed‐rank test for matched pairs. A *p* < 0.05 (95% CI) was considered statistically significant. Effect size was estimated for each significant statistical test with the R correlation coefficient (*r*). Results were considered as small effect size (0.1 ≥ |*r*| ≤ 0.3; slight differences between the two groups), moderate effect size (0.3 ≥ |*r*| ≤ 0.5; medium differences between the two groups) or large effect size (|*r*| ≥ 0.5; high differences between the two groups) [13].

## 3 Results

### 3.1 Participants Baseline Characteristics and General Results

All the subjects (*n* = 8) involved in the trial completed the eight RF sessions and the corresponding assessments. The age range of the participants varied from 35 to 57 years old, with a mean age of 47.63 ± 7.48 years old. Body weight was reduced, on average, from 61.69 ± 11.15 kg at baseline to 60.79 ± 11.65 kg after procedures (Table 1).

**TABLE 1:** Demographic information and clinical results of the RF treatments in the thighs.

Parameter/measurement	Values (*n*)	*p*
Age range (years)	35–57 (*n* = 8)	—
Gender (*n*)		
Female	8	—
Body weight loss (kg; mean ± SD)	0.90 ± 1.28 (*n* = 8)	—
Thighs contours reduction (cm; median ± SD)		
H1	1.80 ± 0.56 (*n* = 7)	0.02*
H2	1.50 ± 0.30 (*n* = 6)	0.03*
Fat layer thickness reduction (%; median ± SD)	20 ± 11.58 (*n* = 13)	0.0016**
Skin echogenicity increase (%; median ± SD)	6 ± 9.40 (*n* = 8)	0.03*

Abbreviations: H1: upper contour; H2: lower contour; *n*: sample size; SD: standard deviation.

* *p* < 0.05.

** *p* < 0.01.

### 3.2 Evaluation of Thighs Circumferences After the Treatments

**FIGURE 1:**
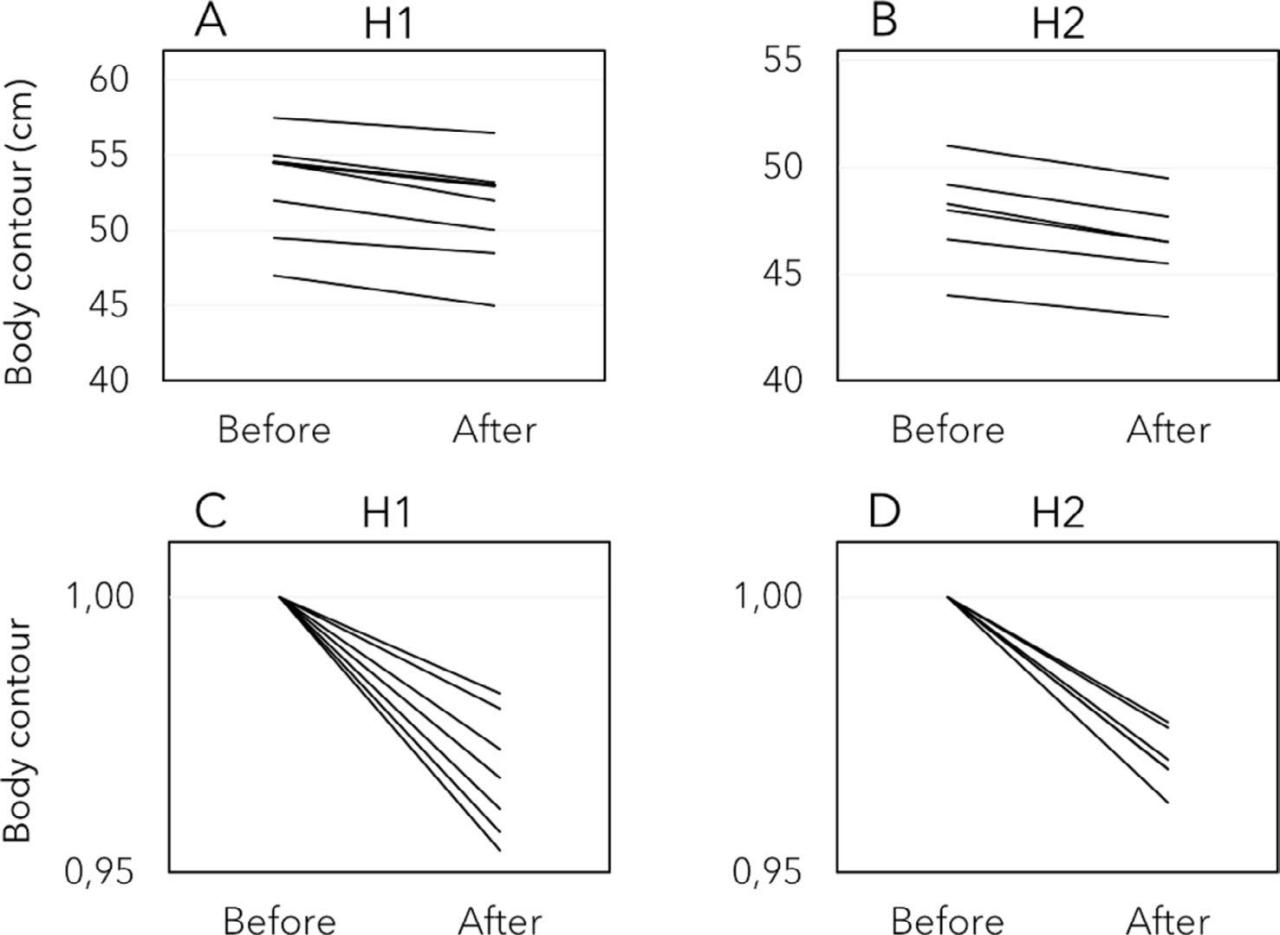
Evaluation of thighs contours following Symmed procedure. Before and after treatment circumferences (cm) at the (A) H1 (*n* = 7) and (B) H2 (*n* = 6) heights. Normalized representations of the reduction at (C) H1 and (D) H2. H1: upper contour; H2: lower contour.

After the treatments, the average body contours in the thighs decreased significantly at the two measuring points (Figure 1). At H1 (upper region of the thigh) a significant 1.80 ± 0 reduction (3%; range: 1–2.50 cm) was observed (*W* = 28; *p* = 0.02; *r* = 0.90). The H2 contour (lower region of the thigh) was significantly reduced (*W* = 21; *p* = 0.03; *r* = 0.91) 1.50 ± 0.30 cm (3%; range: 1–1.80 cm).

### 3.3 Evaluation of Subcutaneous Fat Layer Thickness

**FIGURE 2:**
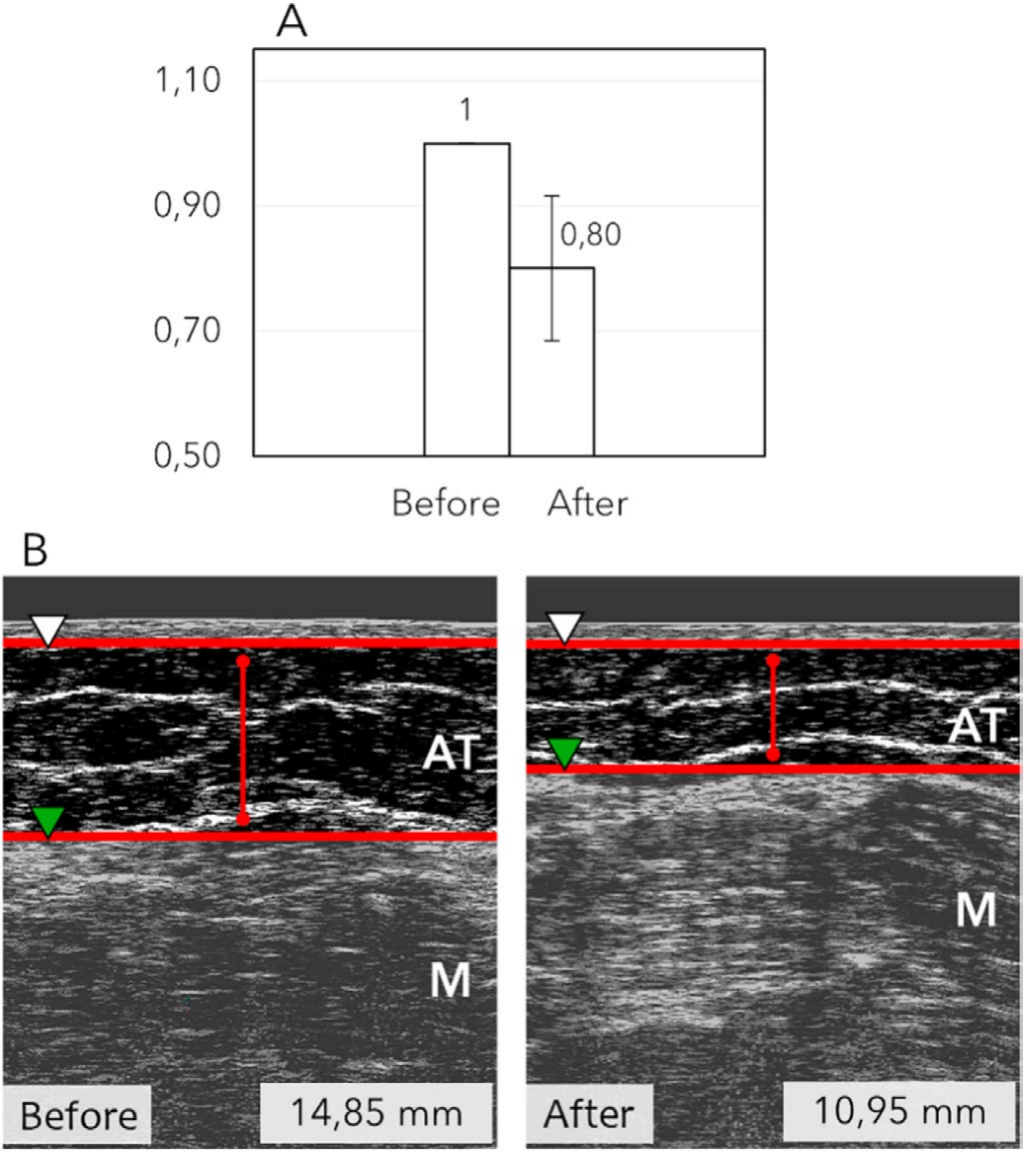
Ultrasound evaluation of subcutaneous fat layer thickness in the thighs after 8 Symmed sessions (*n* = 13). (A) Normalized representations of the average fat layer thickness reduction. (B) Example of subcutaneous ultrasound images showing a fat thickness decrease of 3.90 mm. ▷: deep dermis line; 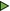: deep fascia membrane; AT: adipose tissue (hypodermis); M: muscle.

The adipose tissue thickness in the thighs was measured as the distance (mm) between the deep dermis line and the deep fascia membrane (Figure 2). After eight sessions, a significant mean reduction of 20% ± 11.58% (2.84 ± 1.47 mm) was noted, with a maximum reduction of 46% (*W* = 91; *p* = 0.0016; *r* = 0.88).

### 3.4 Assessment of DE and Collagen Content

**FIGURE 3:**
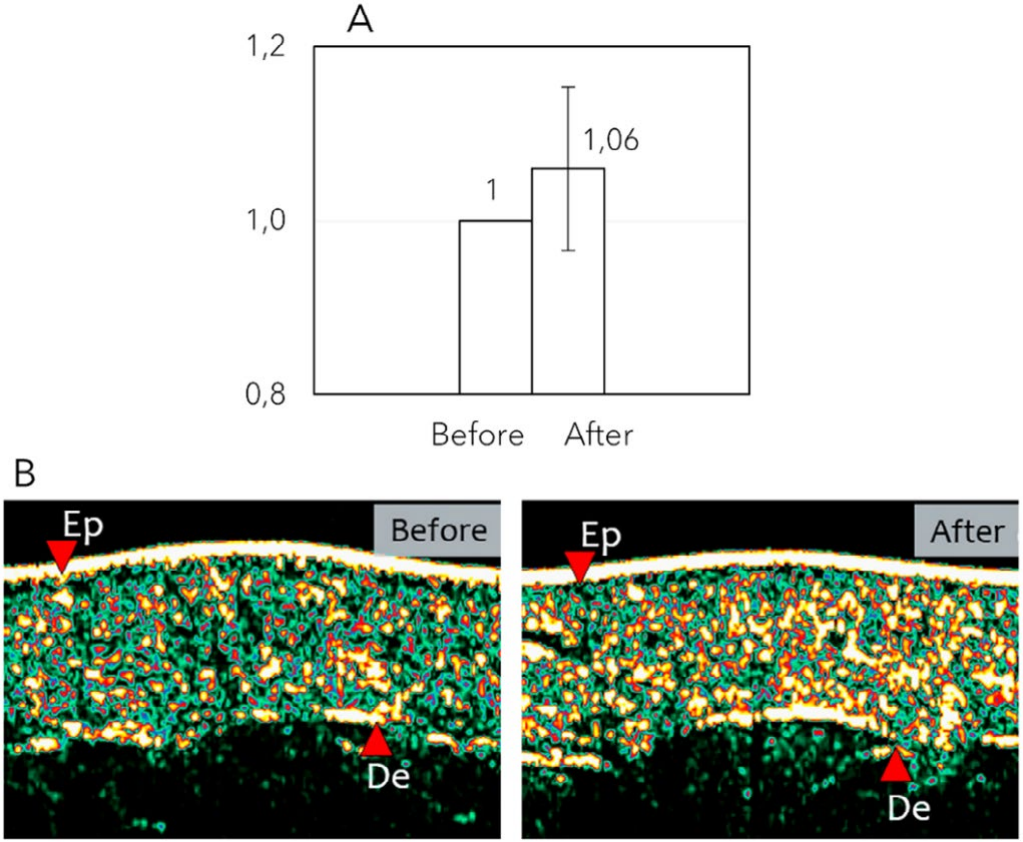
Ultrasound analysis of DE following Symmed treatments (*n* = 8). (A) Normalized representation of DE improvement in the thighs. (B) Skin ultrasound images of a participant showing a DE increase of 21.60% (yellow). Ep: epidermis; De: dermis.

In the study, DE was used as an indirect measurement of the collagen present in the dermis (Figure 3). Following the treatment, DE was increased, on average, by 6% ± 9.40%, with maximum values of 24%. This gain represented a significant improvement in skin echogenicity and, consequently, in dermal collagen content (*W* = 3; *p* = 0.03; *r* = −0.74).

### 3.5 Visual Analysis of Skin Appearance and Texture

**FIGURE 4:**
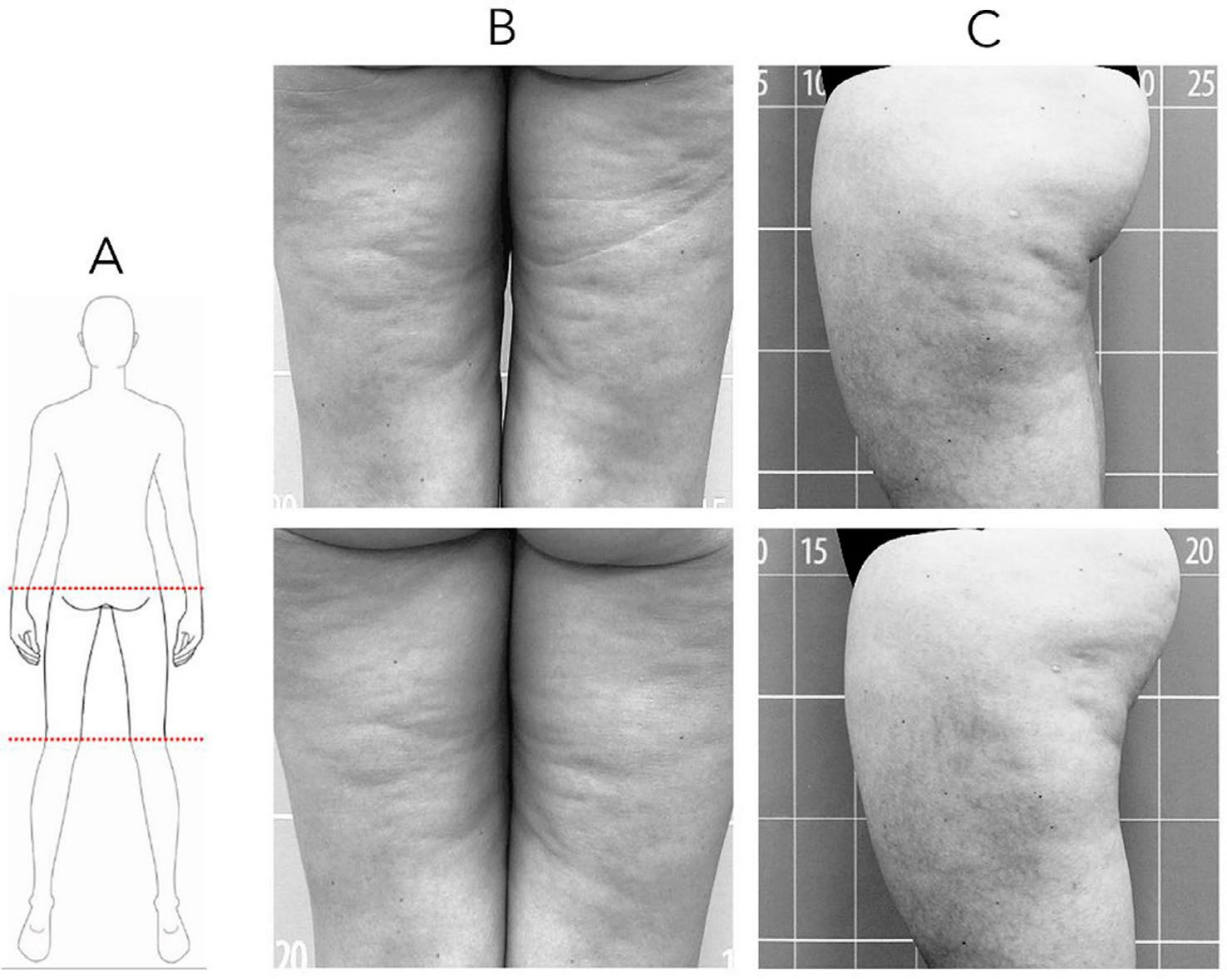
Before (up) and after (down) treatment photographs. (A) Illustration indicating the treated and photographed area. (B, C) Images of two participants exhibiting a reduction in cutaneous rippling and an improvement in the overall skin texture.

Visual evaluation of the RF treatment effectiveness in the thighs was performed by analyzing photographs from different perspectives (Figure 4). A reduction of skin depressions and the rippled appearance of cellulite, as well as an overall improvement in skin texture were observed in a high number of participants. At the follow‐up session a decrease in thigh volume was also noticeable.

### 3.6 Assessment of Adverse Events Occurrence and Satisfaction Questionnaires

Satisfaction surveys were performed by the participants 1 month after finishing the treatments. The questionnaires showed a high level of satisfaction: 100% of the subjects expressed a compliance level between 7 and 10 (on a scale of 0–10). All participants (*n* = 8) also stated they would recommend the same treatment to other people. Treatments were well tolerated, with minimal discomfort in 12.50% (*n* = 1) of the cases resulting from skin heating. No adverse effects were detected after Symmed sessions.

## 4 Discussion

The appearance of skin cellulite and fat tissue changes are two of the most common aesthetic concerns associated with the aging process [14]. Given the high prevalence and the drawbacks associated with these unconformities, it is crucial to effectively ameliorate or eliminate them [11]. However, despite the large number of resources dedicated to it, efforts to develop effective therapies are still ongoing [15]. Besides the effectiveness, in recent years, patients are increasingly searching for safer and non‐invasive treatment options that ensure minimal downtime. Volumetric tissue heating with RF‐based devices has found its application in this area [15].

In this trial, we evaluate the effect of Symmed monopolar RF device for thighs remodeling and skin dermocorrection. Following eight sessions, participants exhibited significant reductions in the (H1) upper and (H2) lower contours of the thighs (H1: 1.80 ± 0.56 cm and H2: 1.50 ± 0.30 cm; 3%). Circumferences measurements were complemented by the ultrasound analysis of subcutaneous fat layer thickness. Results showed an average and significant fat thickness decrease of 2.84 ± 1.47 mm (20%). These findings are in line with the results of clinical studies conducted using devices with Symmed's similar configurations. Yupakorn et al. [16], reported the clinical effect of a monopolar RF device (Indiba, Barcelona, Spain) for the treatment of thighs cellulite. After 10 sessions, they noticed average reductions of 1.84 cm in thighs circumferences and 2.37 mm in subcutaneous tissue thickness. Both observations were statistically significant. Similarly, Albornoz‐Caballero et al. [17], conducted a 10‐sessions study evaluating the efficacy of the Xcultp monopolar device (Biotronic Advance Develops, Granada, Spain) to enhance cellulite condition and reduce the fat layer thickness of the thighs. They obtained significant reductions of thighs circumferences (*p* = 0.01) and fat layer thickness (*p* = 0.028). Finally, Santos et al. [18], found a significant 3% reduction in thighs circumferences using the monopolar Zionic device (Termosalud, Gijón, Spain) during eight sessions. The changes following Symmed RF procedures are also comparable to those noticed with systems combining RF and other energy sources (ultrasound, targeted pressure energy, …). When Fritz et al. [10], combined monopolar RF emission and targeted pressure energy (BTL Unison, BTL Industries, Boston, MA) they detected an average reduction in thighs subcutaneous layer thickness of 1.96 mm.

Following Symmed procedures there was also a significant 6% increase in DE and an overall improvement of dermal structure. Given the high presence of collagen in the dermis, the skin echogenicity parameter is widely used as an indicator of the content and organization of the collagen bundles in this layer [11]. Thus, the dermal hyperechogenic aspect after the treatments indicates an increase in dermal collagen level. Other studies performing ultrasound measurements after RF therapies have also demonstrated this effect. When Mlosek et al. [11], applied a tripolar RF current in the thighs (Beauty Light Science and Technology CO. Ltd., Beijing, China) they also obtained a significant increase (8.92%) in skin echogenicity and, consequently, in the dermal collagen content.

The outcomes following Symmed procedures demonstrate the multilayer stimulation capacity of the device. In a single treatment the system can target dermal and hypodermal tissues and produce beneficial biological changes. Numerous authors have evidenced the importance of this dual effect to treat and improve the occurrence of various age‐related aesthetic problems, such as fat accumulation, skin laxity, and/or cellulite appearance [11, 15, 17, 19]. Nevertheless, because its high prevalence, the latter stands out as one of the most notable concern in the population [20]. Currently, cellulite is a condition affecting between 80% and 90% of post‐pubertal women often identified by the dimpled appearance of the skin's surface [20]. Although the exact causes of its onset are still unclear, it is commonly associated with age, as well as a number of hormonal, hereditary and lifestyle factors that impact in the components and architecture of skin and subcutaneous tissues [7, 15]. In cellulite states it has been suggested that the pressure of accumulated fat (hypertrophic fat cells) over the hypodermic fibrotic septa and the dermo‐hypodermal interface pushes the adipose tissue into the dermis (adipose papillae) creating the dimpled appearance in the surface of the skin [7, 20]. Experimental evidence has revealed that RF treatments generate a decrease in adipocyte fat content and cell volume throughout the stimulation of lipolytic processes (lipolysis) and lipid turnover in the subcutaneous tissue [21, 22]. Thus, the decline in adipocyte size following Symmed RF treatments would decrease the tension into the hypodermal septa and, therefore, the protrusion of fatty tissue towards the dermis [11]. Visually, there would be an improvement in skin topography, with less evidence of the cellulite‐associated rippled appearance [14, 15]. Skin irregularities have been also proposed to be worsened by dermal thinning and atrophy resulting from the degradation and disorganization of the dermal collagen network [19]. In this scenario, RF procedures, through electrothermal stimulation, promote the synthesis of new collagen by fibroblasts [11] and the reorganization of the collagen scaffold that constitutes the ECM [22]. This manner, the effect of RF into the superficial tissues contributes to reconstitute the architecture of the dermis, attenuate the alterations of the skin surface and, consequently, enhance its visual appearance [11].

Besides the mentioned causes, both the appearance of cellulite and the fat accumulation have an important vascular component [7, 19]. In both conditions there is a local dysfunction of blood circulation and lymphatic drainage patterns, leading to fluid retention in the spaces between the fat lobules and inter‐ lobular septa of the affected areas [19, 23]. In this regard, the application of physical massage has its relevance [24]. Together with RF, treatments using the Symmed device involve a mechanical massage produced by the manual movement of the electrodes on the skin. Studies evaluating the effect of these massages in aesthetic procedures demonstrate its effectiveness decreasing accumulated tissues fluids, eliminating waste substances, as well as, reducing the thickness of the fat layer [23, 24]. Thus, the performance of the mechanical massage in our study potentiates the improvement of the appearance of the skin and the body remodeling effect detected when applying RF.

The effects described in this investigation provide evidence for the safety and effectiveness of Symmed RF device in cellulite and body‐remodeling treatments. Given the inclusion of people up to 57 years of age, this clinical trial also constitutes a valuable contribution to the evaluation of strategies to combat cellulite exacerbation in older people. Numerous investigations have been focused on the assessment of cellulite RF treatments in patients from 18 to 40 years old [10, 17, 22], when the aesthetic concern becomes noticeable, and patients most commonly seek for treatment options [25]. However, the significant deterioration of cellulite status in older age groups (from 50 years old) as a consequence of hormonal imbalances related to aging and possible hormonal replacement therapies also makes these groups a target population to determine the effectiveness of RF procedures [25]. Additionally, the evaluation of these treatments can be performed using different methodologies, usually including ultrasound assessment of the subcutaneous tissues [3, 17]. In our investigation fat layer thickness analysis is complemented with the ultrasound study of DE and thereby of the content of collagen in tissue's fibrous septa [11]. As has been previously mentioned, both factors are directly involved in the worsening of cellulite condition. Thus, its combined evaluation increases the knowledge of RF effect in cellulite condition treatments. Considering these elements, the results of our investigation demonstrated that dermal and adipose tissue were stimulated leading to the improvement of skin properties and the reduction of fat tissue and body circumferences. Further trials will be considered to quantify the improvement in a larger number of patients and to histologically detail the changes produced in relation to the reduction of adipose tissue thickness and dermal collagen content. However, our findings prove Symmed is a reliable option in the non‐invasive treatment of skin imperfections and improvement of skin appearance.

## 5 Conclusion

Symmed RF application is an effective, safe and tolerable treatment to improve the appearance of thighs cellulite appearance by reducing adipose tissue layer thickness and increasing dermal collagen content.

## Author Contributions

All authors contributed to the clinical research performance and approved the final content of the article. **A.I.F and L.S.F:** volunteers' recruitment, scheduling, treatment performance and safety supervising. **A.F.S, L.H.Z and S.V.F :** recruitment, study design, research monitoring, acquisition, analysis and interpretation of data and manuscript drafting.

## Ethics Statement

The project was approved by the Research Ethics Committee of the Principality of Asturias with the registration number 2022.340 on September 22nd, 2022. It adheres to the principles established by the Declaration of Helsinki (1964) and its later amendments. Prior to the start of the study all subjects signed written informed consent for participation in the trial and the use of its photographs.

## Conflicts of Interest

The research team for this study was composed by Termosalud Inc. (Gijón, Spain) personnel. All the treatments were performed in a center associated to the company. The study was supervised by the Asturias Research Ethics Committee and an authorized Clinical Research Associate (CRA) to ensure the correct development of the research and data analysis. The authors of the paper are responsible for the content of the article.

## Supporting information


File S1.

## Data Availability

The data that support the findings of this study are available from the corresponding author upon reasonable request.
