# Supplementary material for: Effectiveness of Body Remodeling and Cellulite Appearance Improvement Treatments in the Thighs Using Symmed Radiofrequency Device
Source: J Cosmet Dermatol. 2025 Jan 15;24(1):e16796. doi: 10.1111/jocd.16796 (PMC11735865; doi:10.1111/jocd.16796)
Supplement: Supplementary file 1 — File S1. [file JOCD-24-e16796-s001.docx]

**SUPPLEMENTARY MATERIALS**

**Supplementary material S1.- Symmed^TM^ Contraindications**

- Pregnancy and lactation. Pregnancy is a contraindication in the application of radiofrequency treatments, so you should not be pregnant at the time of starting the treatment and you should avoid becoming pregnant during the whole period in which the treatments are performed

- Coagulation disorders, or anticoagulant medications

- Uncontrolled hypertension

- Diabetes mellitus

- Phlebitis or vasculitis

- Cancer or tumors

- Obesity

- Vascular fragility

- Recent surgery (6 weeks)

- Infections, irritated or eroded areas or open wounds on the skin in the area to be treated

- Varicose veins (in the treatment area)

- Medical conditions such as: heart, lung or circulatory disease

- Inability to understand the consequences, implications, and risks of the procedure

- Elevated body temperature (pyrexia)

- Presence of metallic implants or prostheses in the area.

- Presence of metallic IUD (in case the treatment is performed in the abdomen).

- Pacemaker wearers

- Medication

- Concomitant devices.

**Supplementary material S2.- Patient flow diagram**


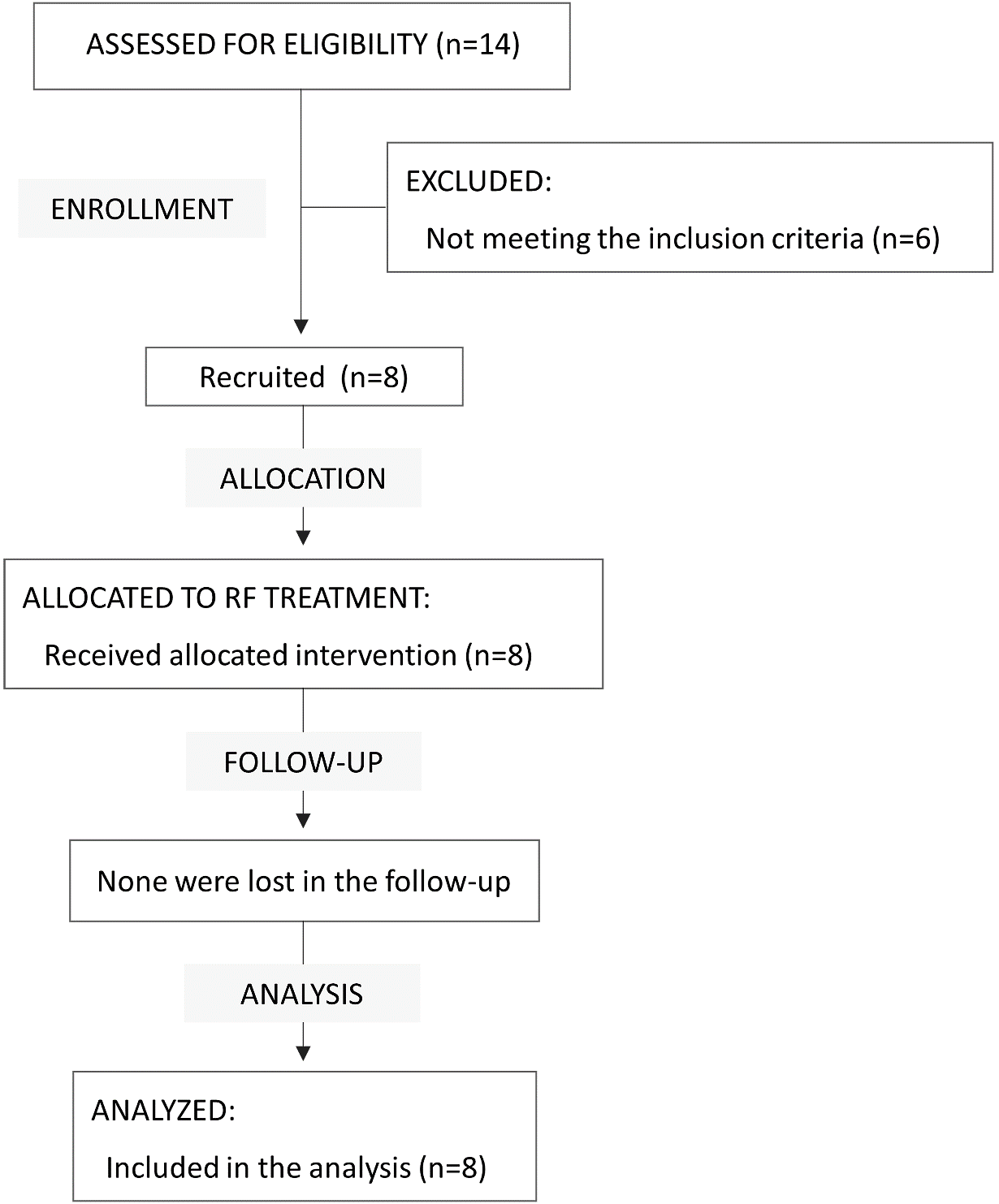


Figure. Flowchart of the subjects involved in the clinical trial (CONSORT).

**Supplementary material S3.- Clinical data**

Table. Number of measurements taken in the thighs. These measurements were made at baseline and repeated after the last session.

|  | Thighs  (n) |
| --- | --- |
| Body contours | 16 |
| H1 | 8 |
| H2 | 8 |
| Photographs | 32 |
| Skin echogenicity | 24 |
| Fat layer thickness | 24 |

H1: upper contour; H2: lower contour.

n: number of measurements.

**Supplementary material S4.- Anatomical localization of measuring sites**


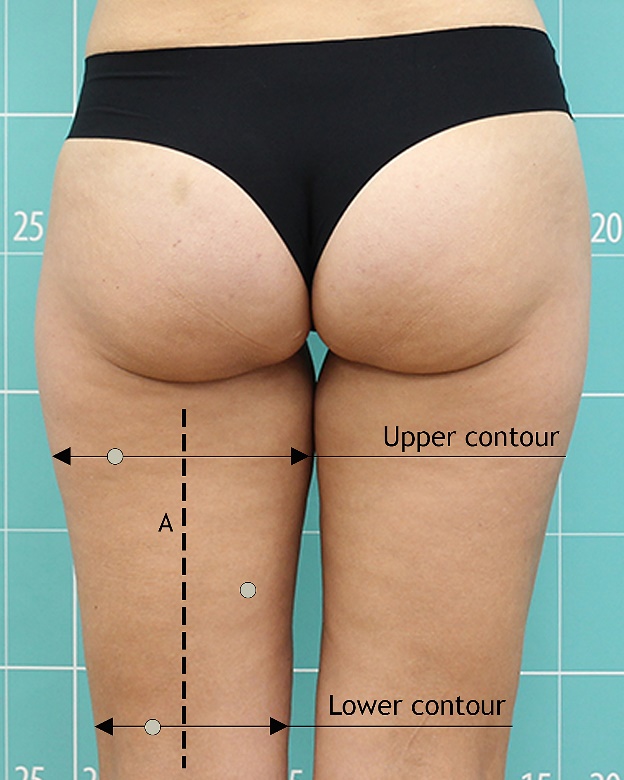


Figure. Anatomical localization of the 2 body contours ( ) measuring heights and the 3 ultrasound measuring points ( ) in the thighs. Coordinates of each site were noted as height from the floor and distance from an anatomical reference axis (A). A: popliteal line.

**Supplementary material S5.- Satisfaction survey**

1. Indicate your gender.

| Man | Woman | Other (specify) |
| --- | --- | --- |

1. Indicate your age.

| <25  26-35  36-45 | 46-55  56-65  > 65 |
| --- | --- |

1. What was the reason you underwent this treatment? You may indicate more than one option.

| Localized fat reduction | Body contour remodeling | Anti-cellulite |
| --- | --- | --- |

1. In what area was the treatment performed? You may indicate more than one option.

| Abdomen  Flanks  Thighs | Buttocks  Other. Specify where. |
| --- | --- |

1. From 1 to 10, how satisfied are you with the results obtained after the treatment with Symmed^TM^ device?

1 2 3 4 5 6 7 8 9 10

1. From 1 to 10, how do you valorate the attention received from the treatment performers?

1 2 3 4 5 6 7 8 9 10

1. From 1 to 10, how satisfied do you feel with the information provided about the treatment performance prior to its beginning?

1 2 3 4 5 6 7 8 9 10

1. Did you experience any of the following adverse event? Mark the corresponding ones.

| Pain  Erythema (skin redness)  Burns | Allergy  Bruising  Blood pressure drop  Other. Specify which one. |
| --- | --- |

1. If you have suffered discomfort during the performance of the treatment, how would you rate it from 1 to 10?

1 2 3 4 5 6 7 8 9 10

1. Would you recommend the treatment to other people?

Yes No

1. When did you begin to notice the positive effects of the treatment?

After the first session After the fifth session

After the second session After the sixth session

After the third session After the seventh session

After the fourth session After the eighth session

Other (describe)

1. Had you previously heard of the capacitive and resistive radiofrequency technique?
2. What were your expectations of the treatment prior to its beggining?
